# Supplementary figures and images for: SLE serum induces altered goblet cell differentiation and leakiness in human intestinal organoids
Source: EMBO Mol Med. 2024 Feb 5;16(3):7. doi: 10.1038/s44321-024-00023-3 (PMC10940301; doi:10.1038/s44321-024-00023-3)

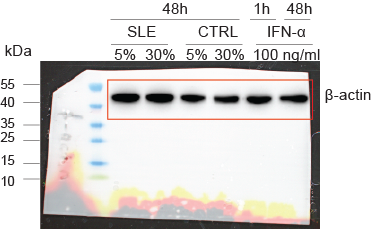

Supplement: Supplementary file 13 — Source Data Fig. 2 [file 44321_2024_23_MOESM13_ESM.zip › Figure 2/2D/Figure_2D_Western_blot_bactin_label.png]

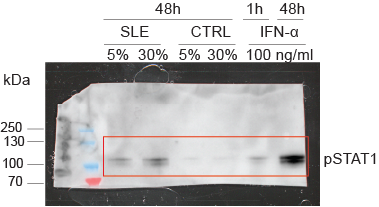

Supplement: Supplementary file 13 — Source Data Fig. 2 [file 44321_2024_23_MOESM13_ESM.zip › Figure 2/2D/Figure_2D_Western_blot_pSTAT1_label.png]

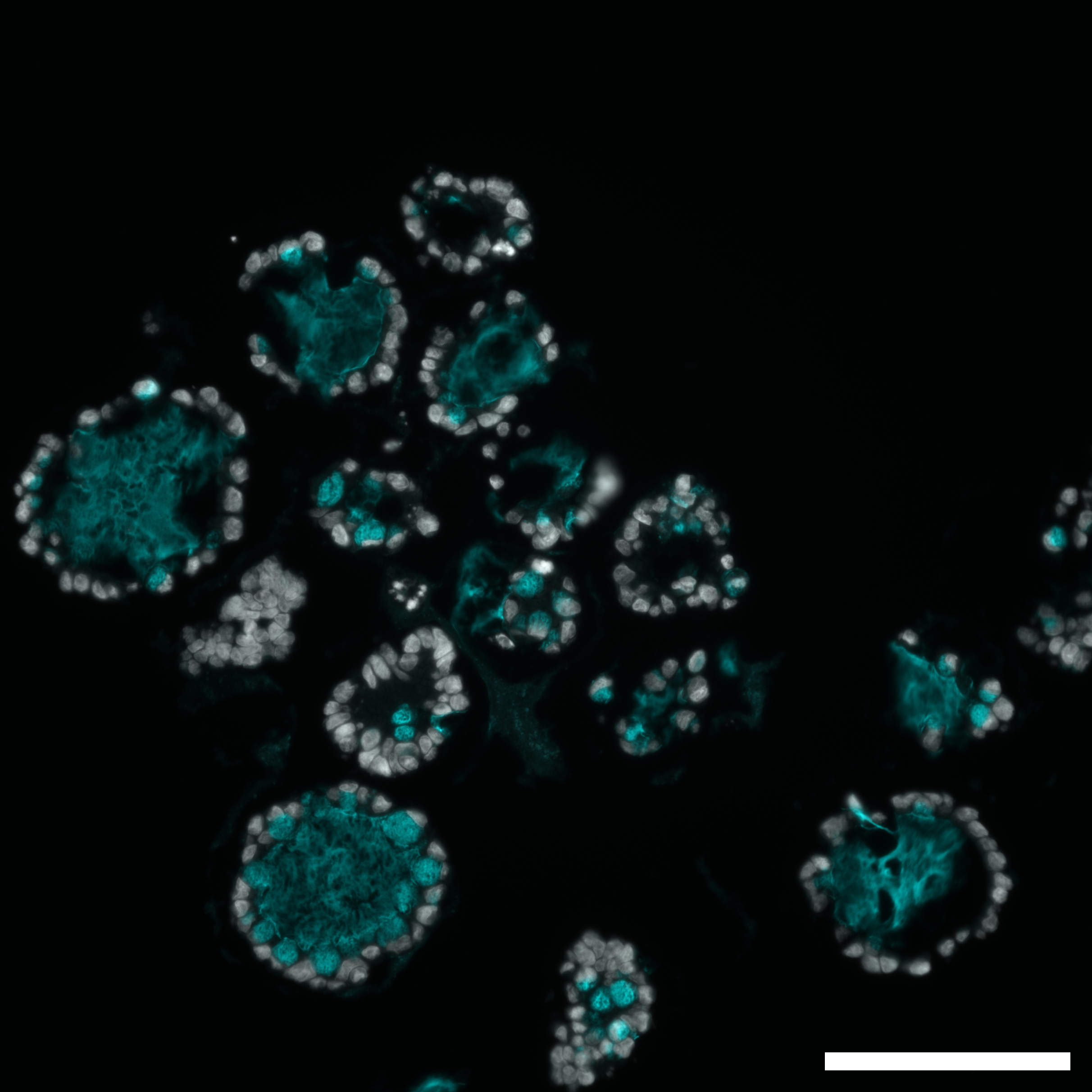

Supplement: Supplementary file 15 — Source Data Fig. 4 [file 44321_2024_23_MOESM15_ESM.zip › Figure 4/4E/Source images 4E/CTRL_composite.tif]

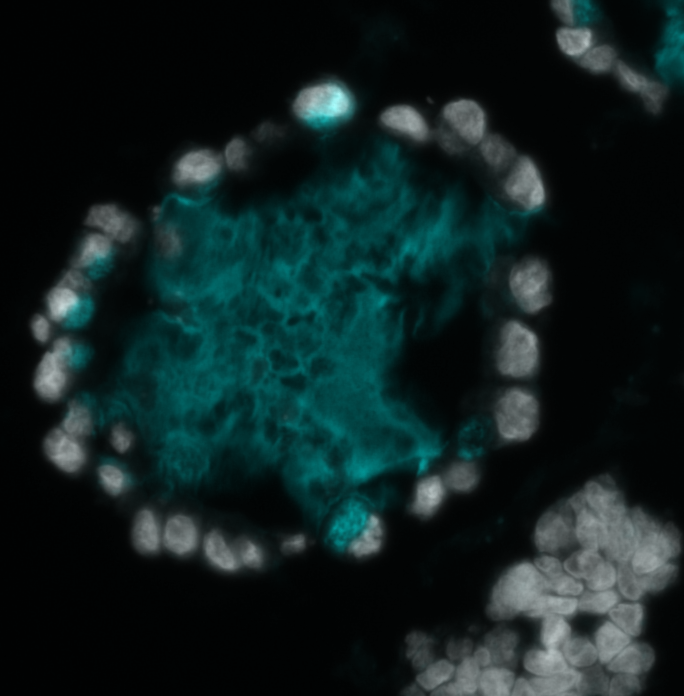

Supplement: Supplementary file 15 — Source Data Fig. 4 [file 44321_2024_23_MOESM15_ESM.zip › Figure 4/4E/Source images 4E/CTRL_composite_zoom.tif]

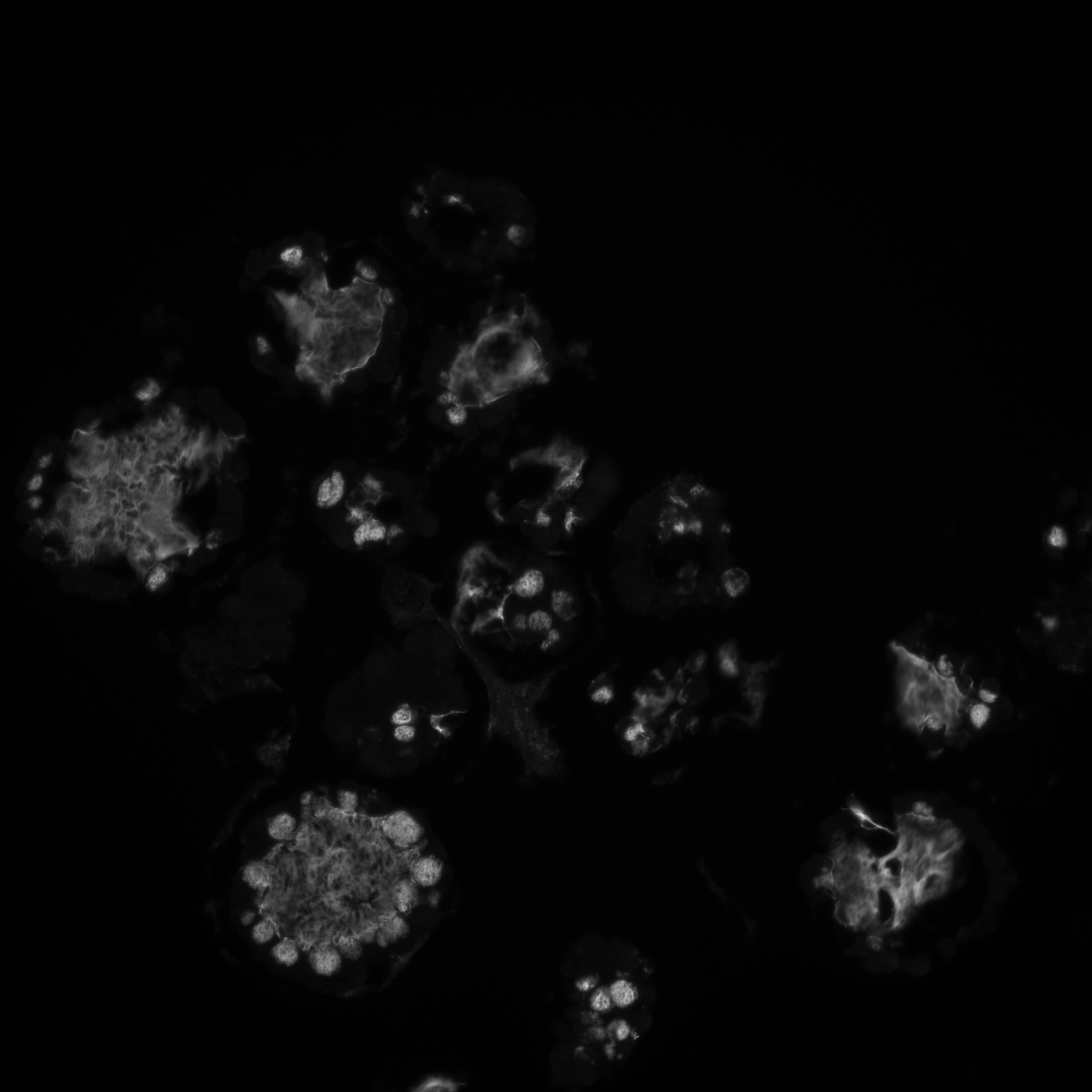

Supplement: Supplementary file 15 — Source Data Fig. 4 [file 44321_2024_23_MOESM15_ESM.zip › Figure 4/4E/Source images 4E/CTRL_MUC2.tif]

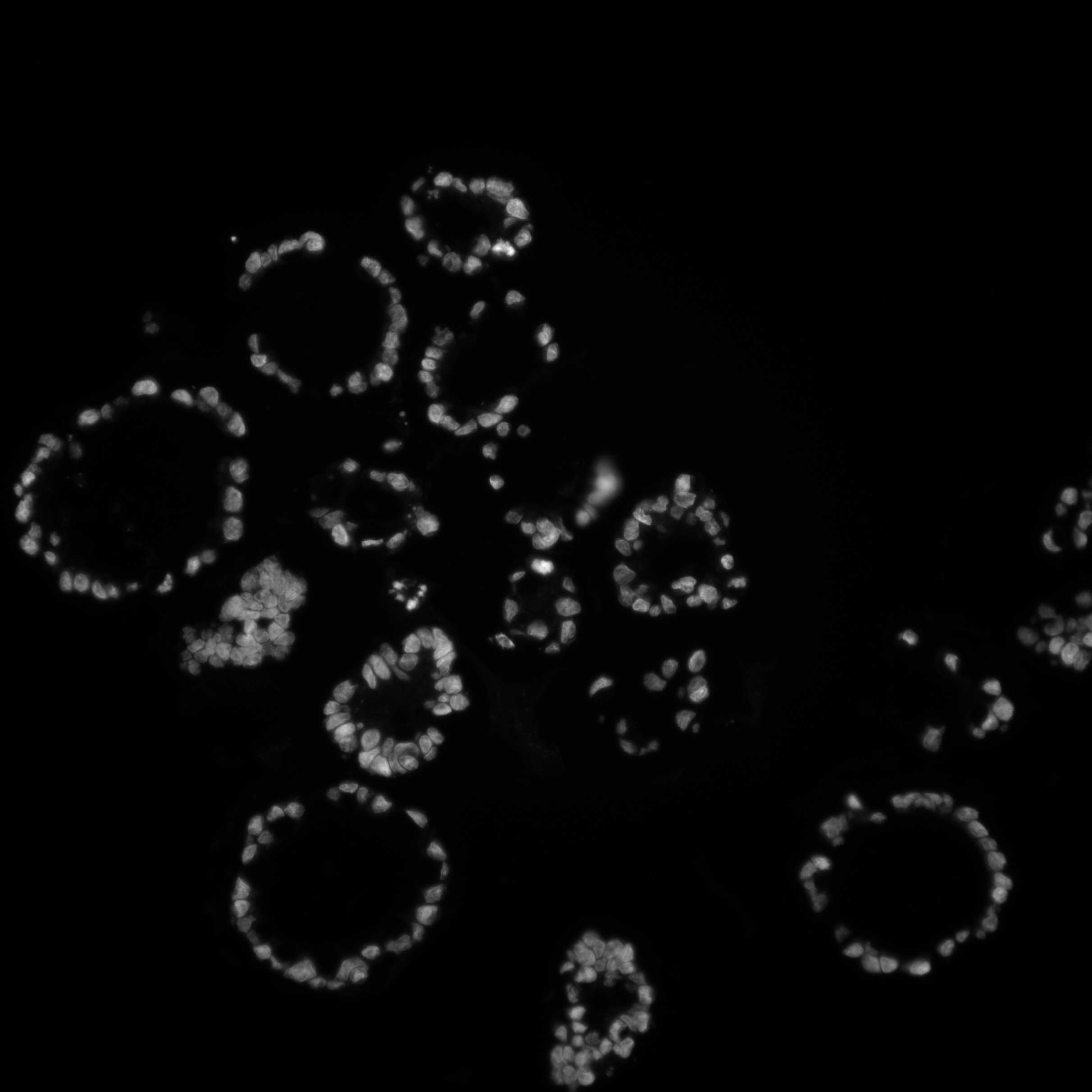

Supplement: Supplementary file 15 — Source Data Fig. 4 [file 44321_2024_23_MOESM15_ESM.zip › Figure 4/4E/Source images 4E/CTRL_nucleus.tif]

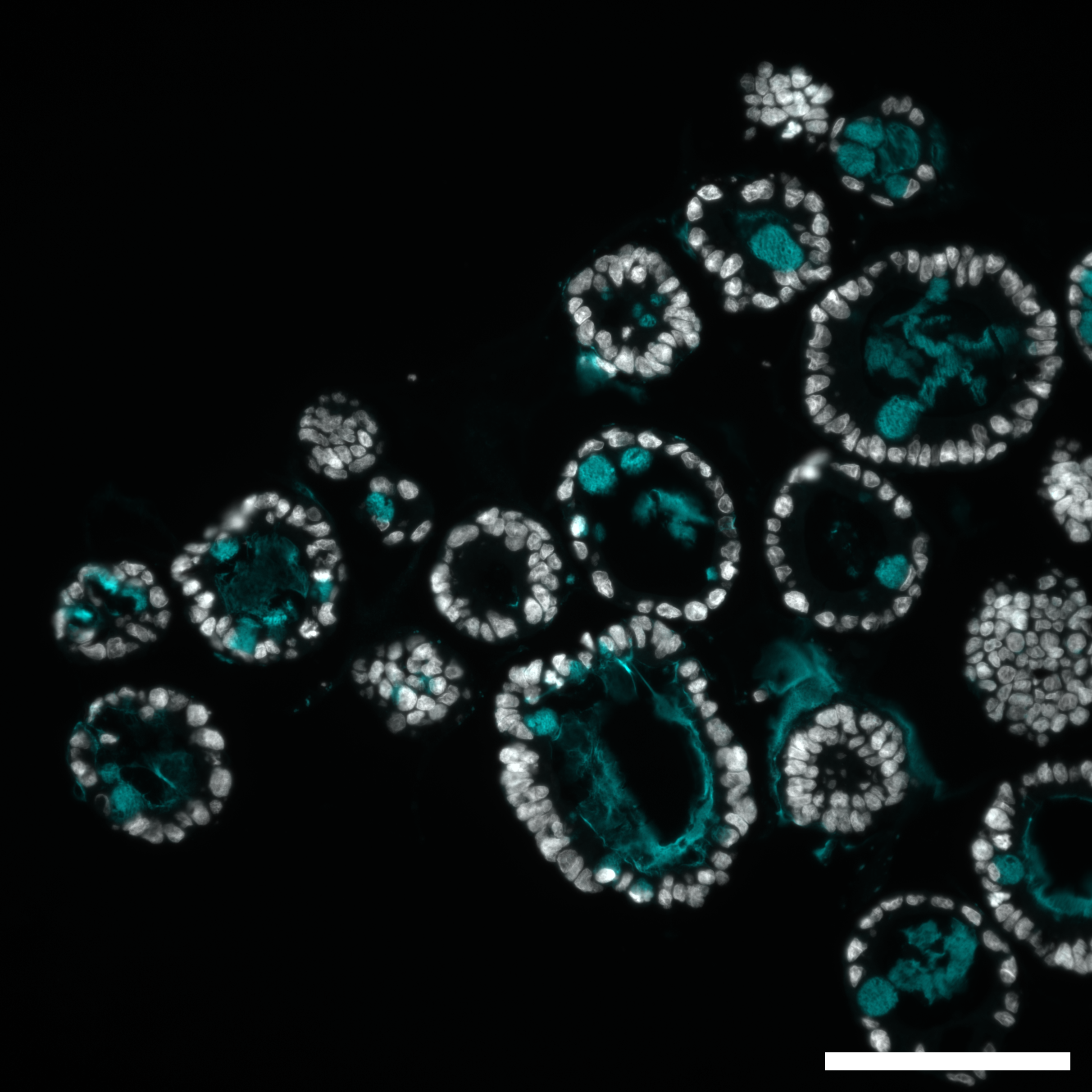

Supplement: Supplementary file 15 — Source Data Fig. 4 [file 44321_2024_23_MOESM15_ESM.zip › Figure 4/4E/Source images 4E/SLE_composite.tif]

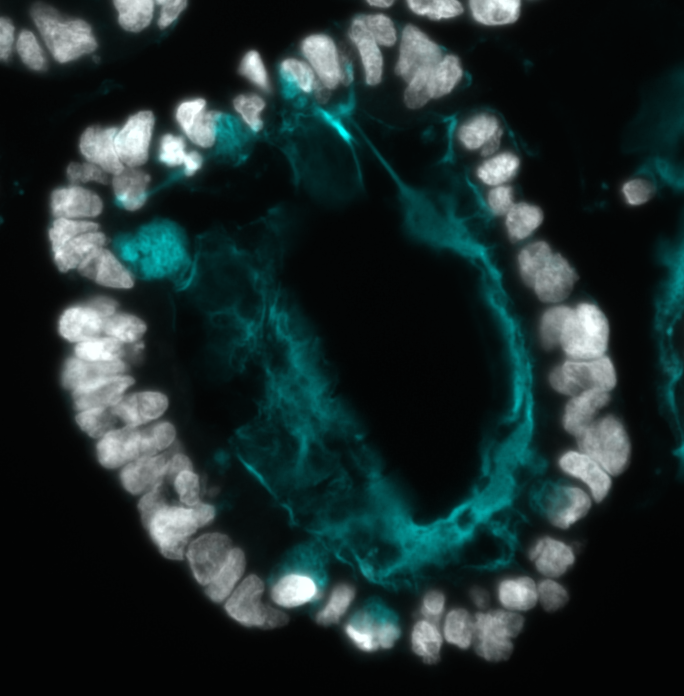

Supplement: Supplementary file 15 — Source Data Fig. 4 [file 44321_2024_23_MOESM15_ESM.zip › Figure 4/4E/Source images 4E/SLE_composite_zoom.tif]

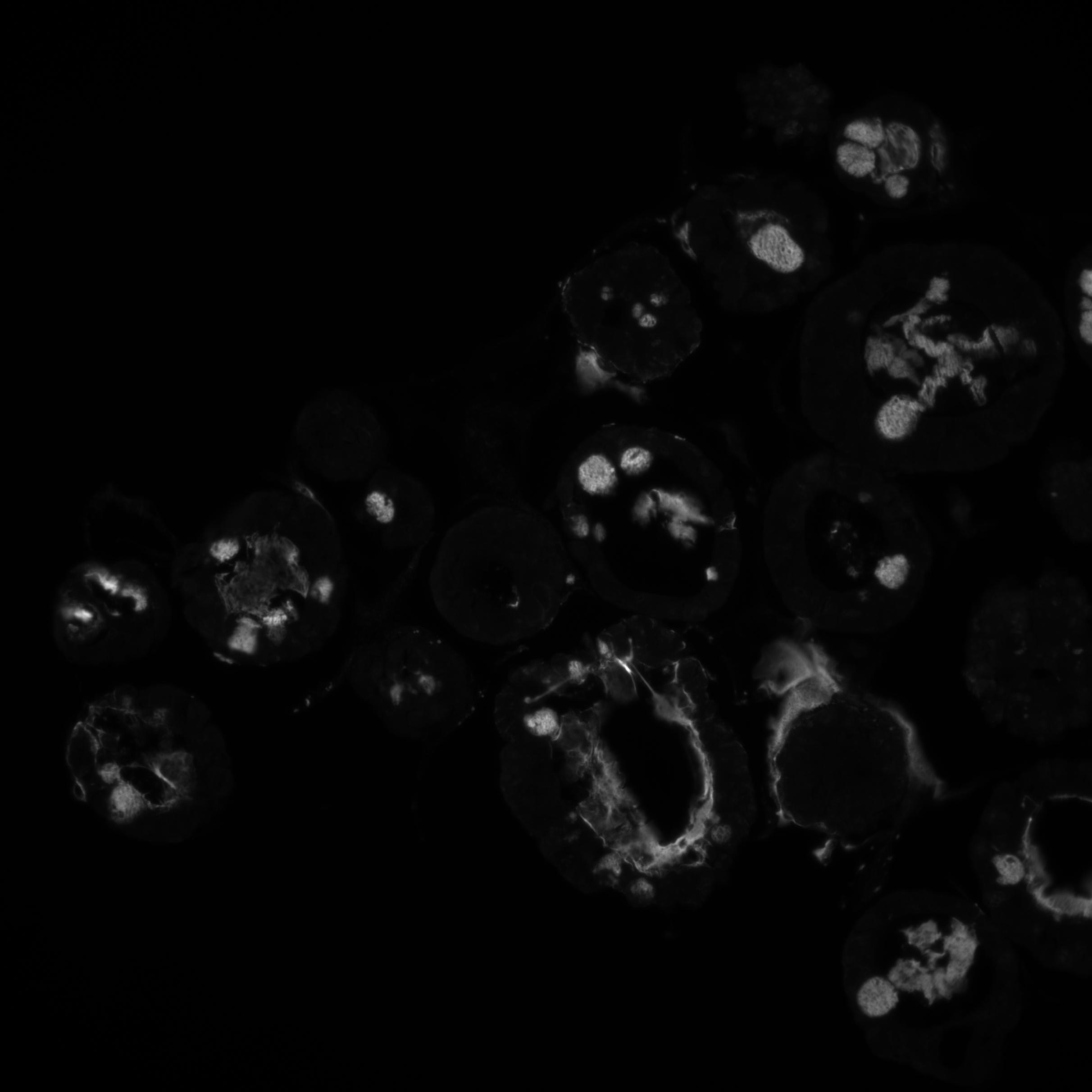

Supplement: Supplementary file 15 — Source Data Fig. 4 [file 44321_2024_23_MOESM15_ESM.zip › Figure 4/4E/Source images 4E/SLE_MUC2.tif]

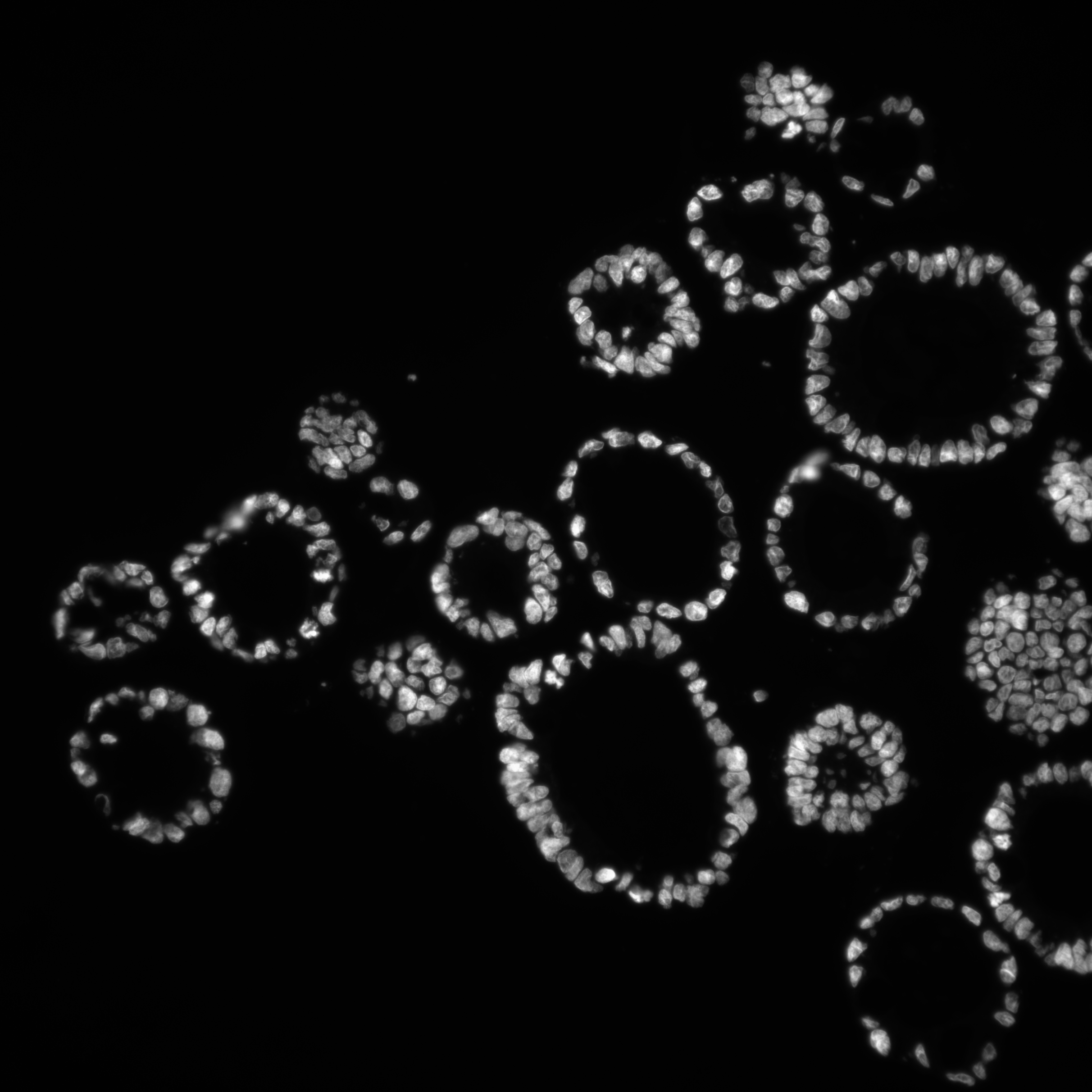

Supplement: Supplementary file 15 — Source Data Fig. 4 [file 44321_2024_23_MOESM15_ESM.zip › Figure 4/4E/Source images 4E/SLE_nucleus.tif]

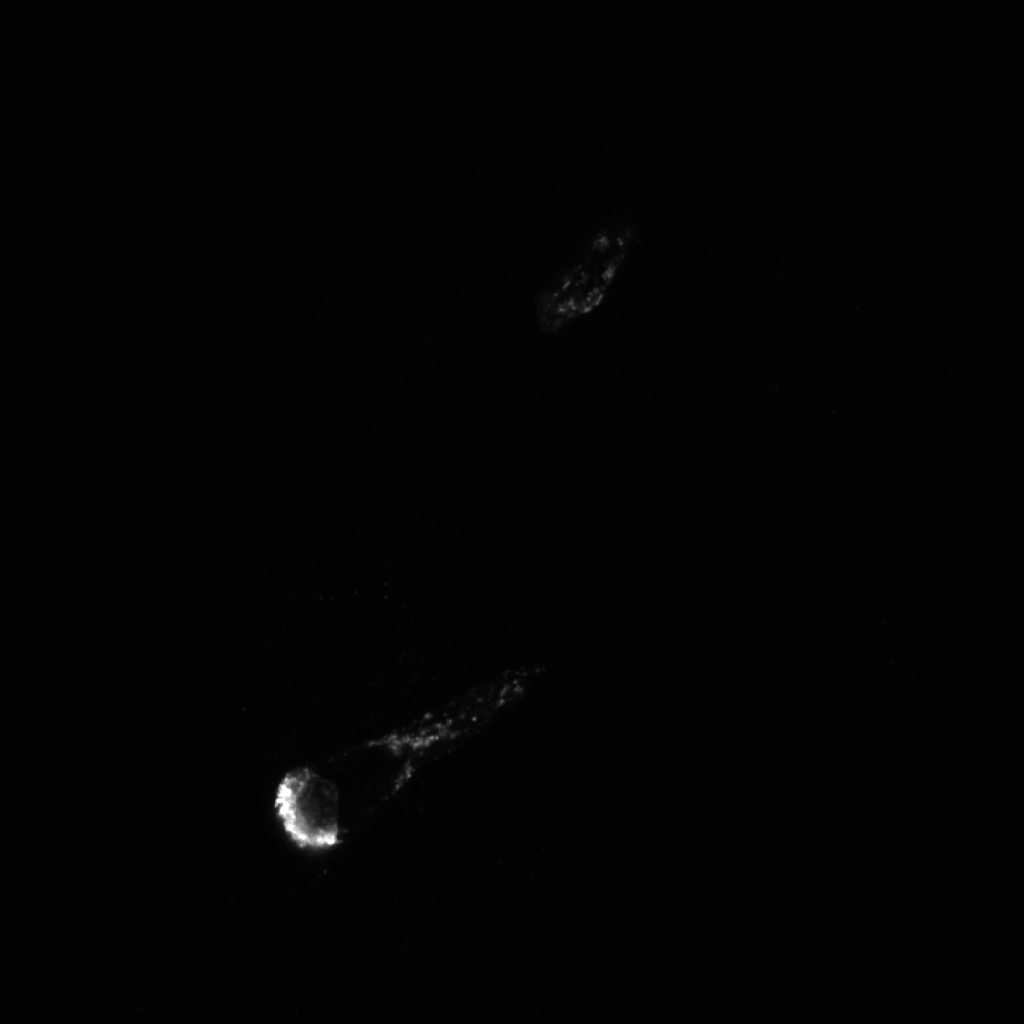

Supplement: Supplementary file 15 — Source Data Fig. 4 [file 44321_2024_23_MOESM15_ESM.zip › Figure 4/4H/Source Images 4H/CTRL_CHGA.tif]

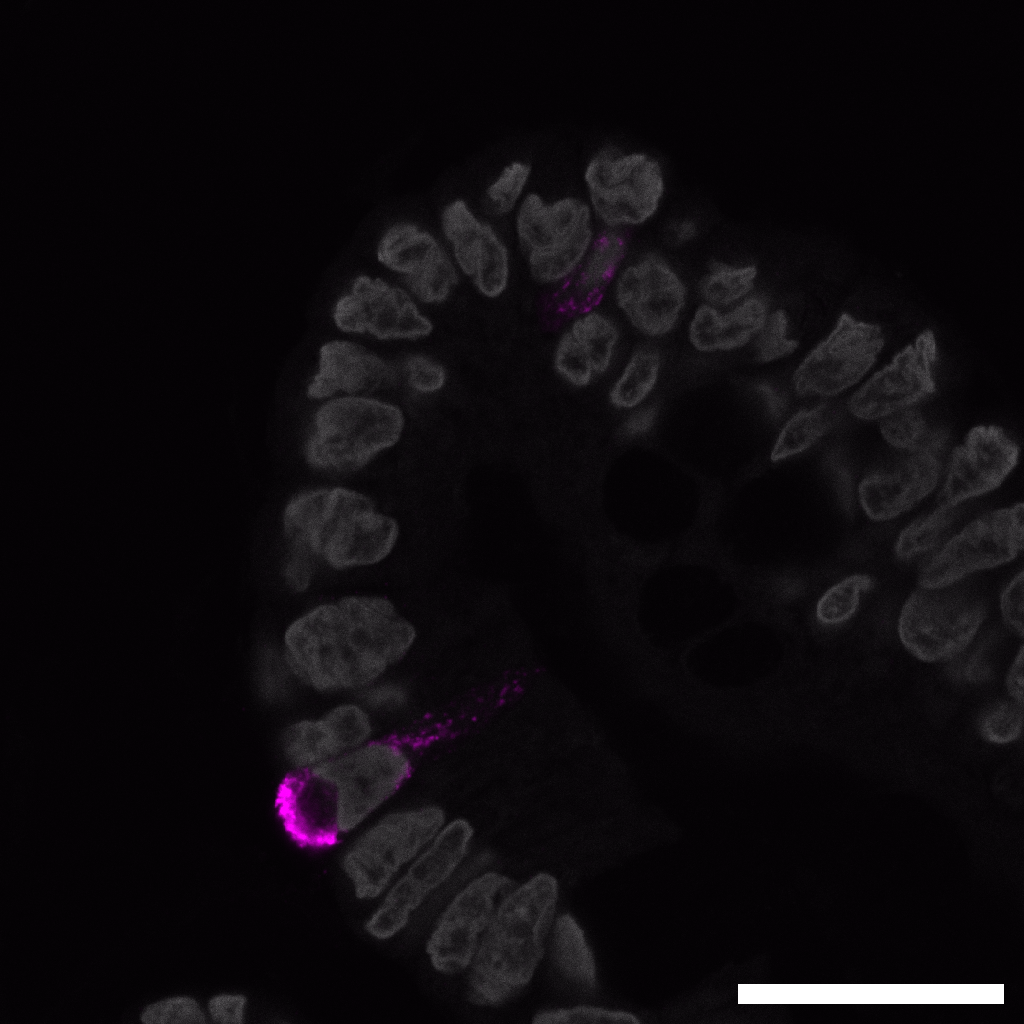

Supplement: Supplementary file 15 — Source Data Fig. 4 [file 44321_2024_23_MOESM15_ESM.zip › Figure 4/4H/Source Images 4H/CTRL_composite.tif]

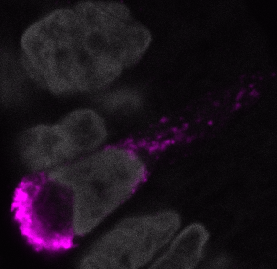

Supplement: Supplementary file 15 — Source Data Fig. 4 [file 44321_2024_23_MOESM15_ESM.zip › Figure 4/4H/Source Images 4H/CTRL_composite_zoom.tif]

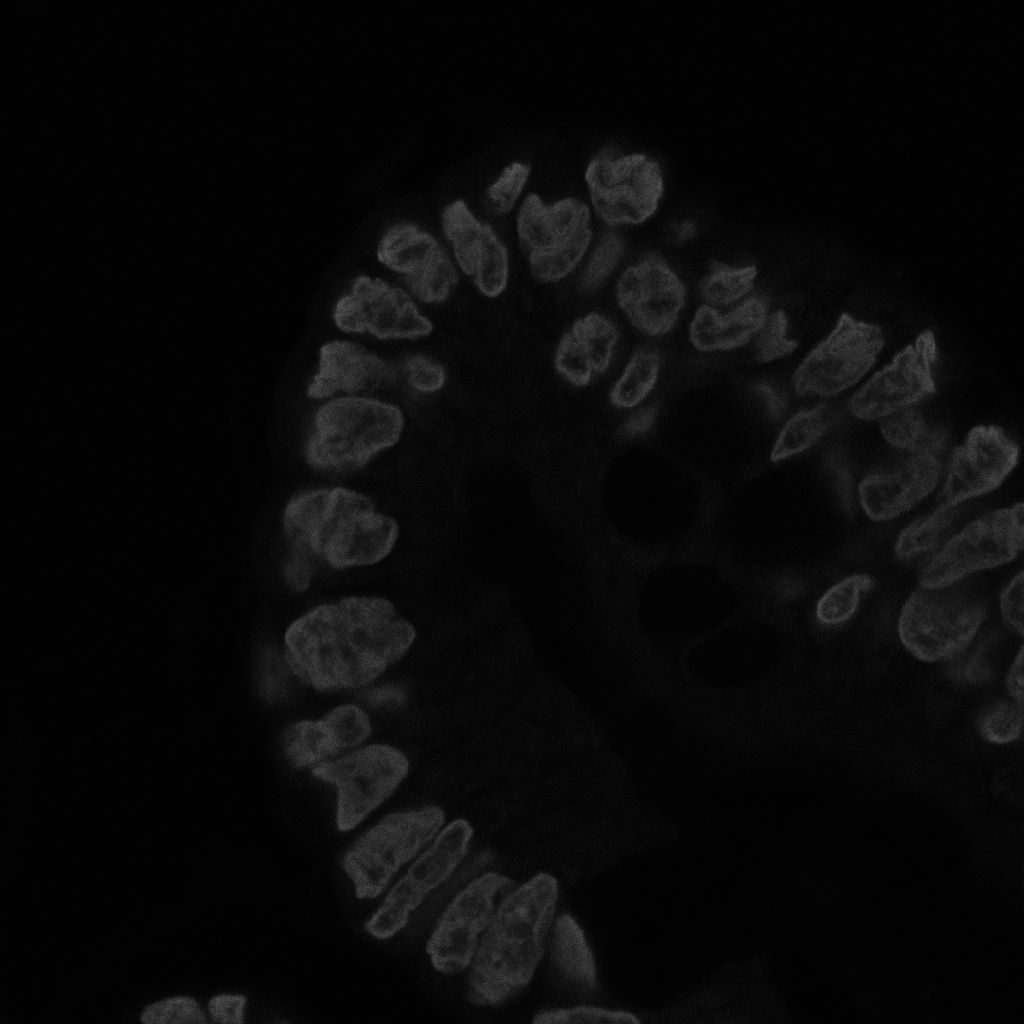

Supplement: Supplementary file 15 — Source Data Fig. 4 [file 44321_2024_23_MOESM15_ESM.zip › Figure 4/4H/Source Images 4H/CTRL_nucleus.tif]

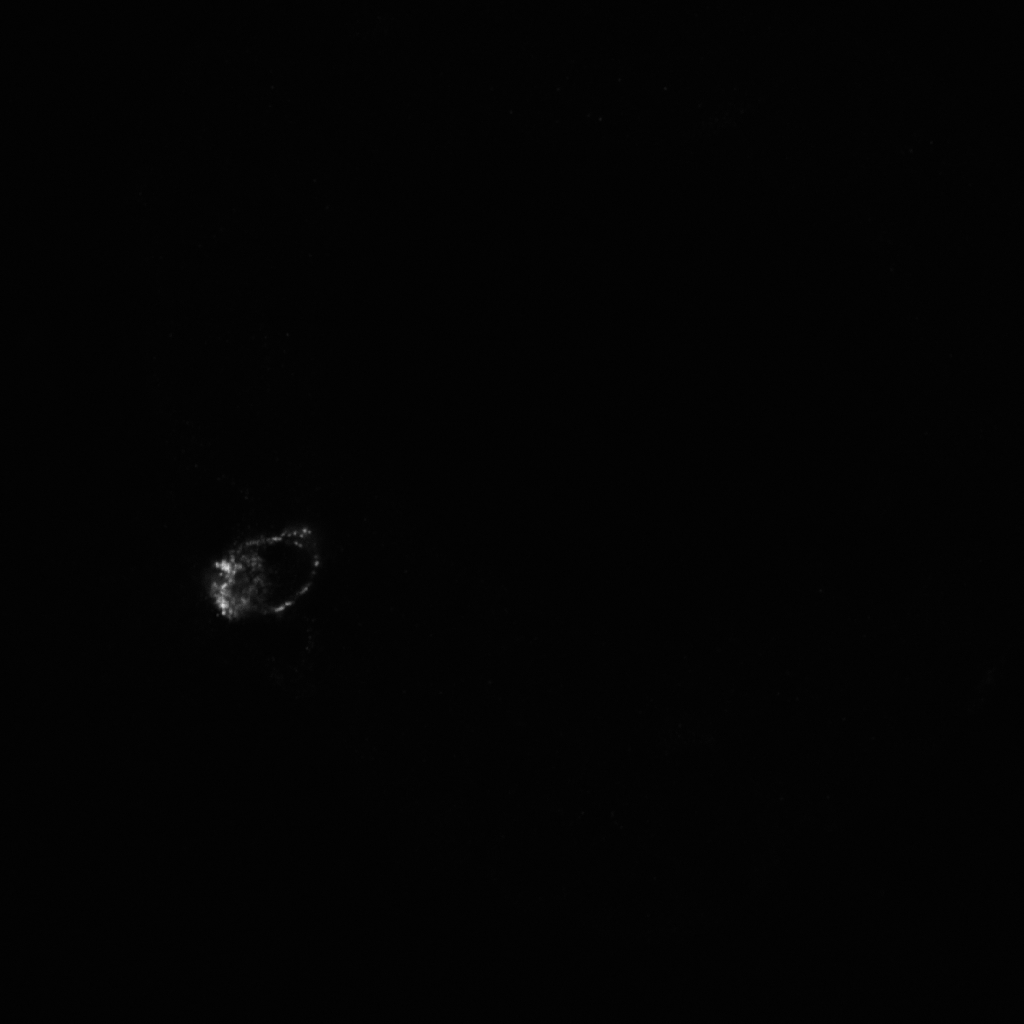

Supplement: Supplementary file 15 — Source Data Fig. 4 [file 44321_2024_23_MOESM15_ESM.zip › Figure 4/4H/Source Images 4H/SLE_CHGA.tif]

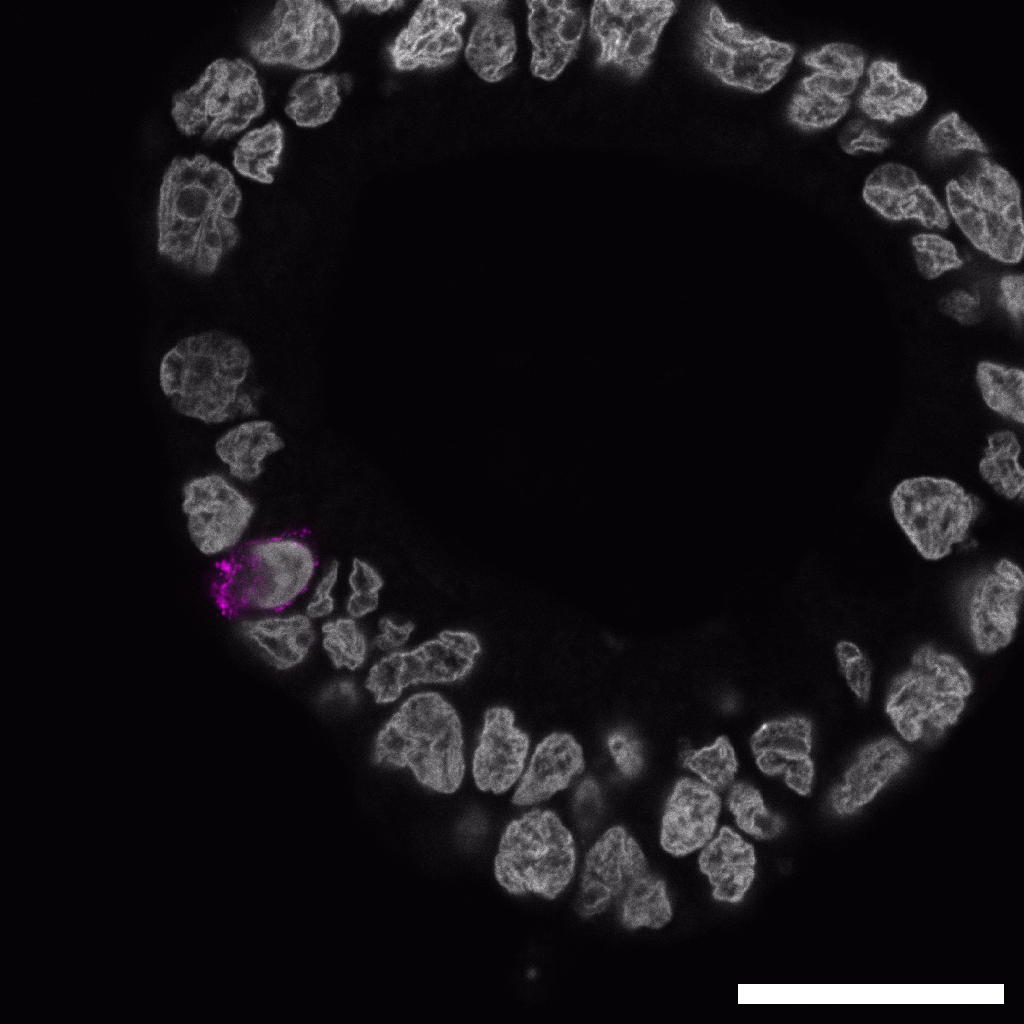

Supplement: Supplementary file 15 — Source Data Fig. 4 [file 44321_2024_23_MOESM15_ESM.zip › Figure 4/4H/Source Images 4H/SLE_composite.tif]

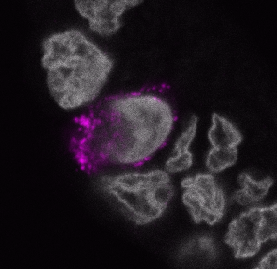

Supplement: Supplementary file 15 — Source Data Fig. 4 [file 44321_2024_23_MOESM15_ESM.zip › Figure 4/4H/Source Images 4H/SLE_composite_zoom.tif]

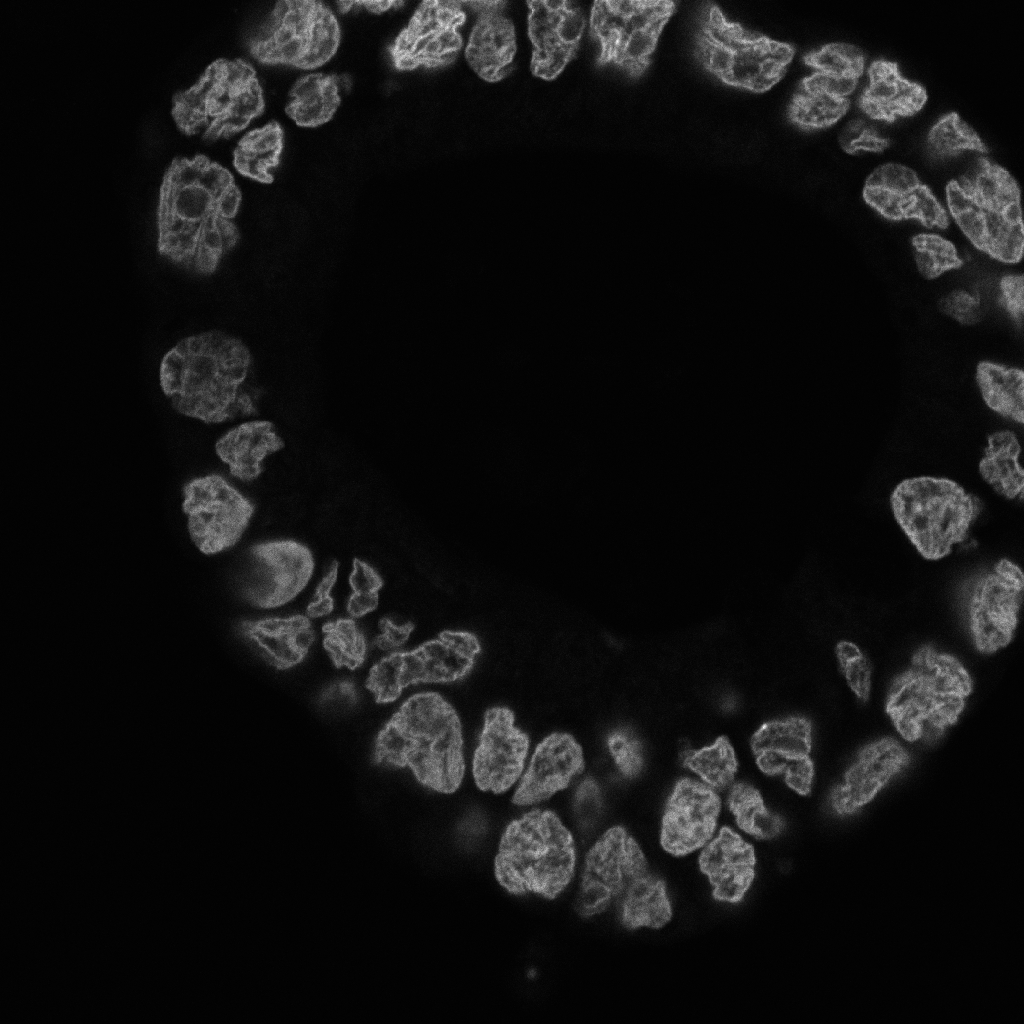

Supplement: Supplementary file 15 — Source Data Fig. 4 [file 44321_2024_23_MOESM15_ESM.zip › Figure 4/4H/Source Images 4H/SLE_nucleus.tif]
