# Supplementary material for: SLE serum induces altered goblet cell differentiation and leakiness in human intestinal organoids
Source: EMBO Mol Med. 2024 Feb 5;16(3):7. doi: 10.1038/s44321-024-00023-3 (PMC10940301; doi:10.1038/s44321-024-00023-3)
Supplement: Supplementary file 18 — Expanded View Figures [file 44321_2024_23_MOESM18_ESM.pdf]

Expanded View Figures

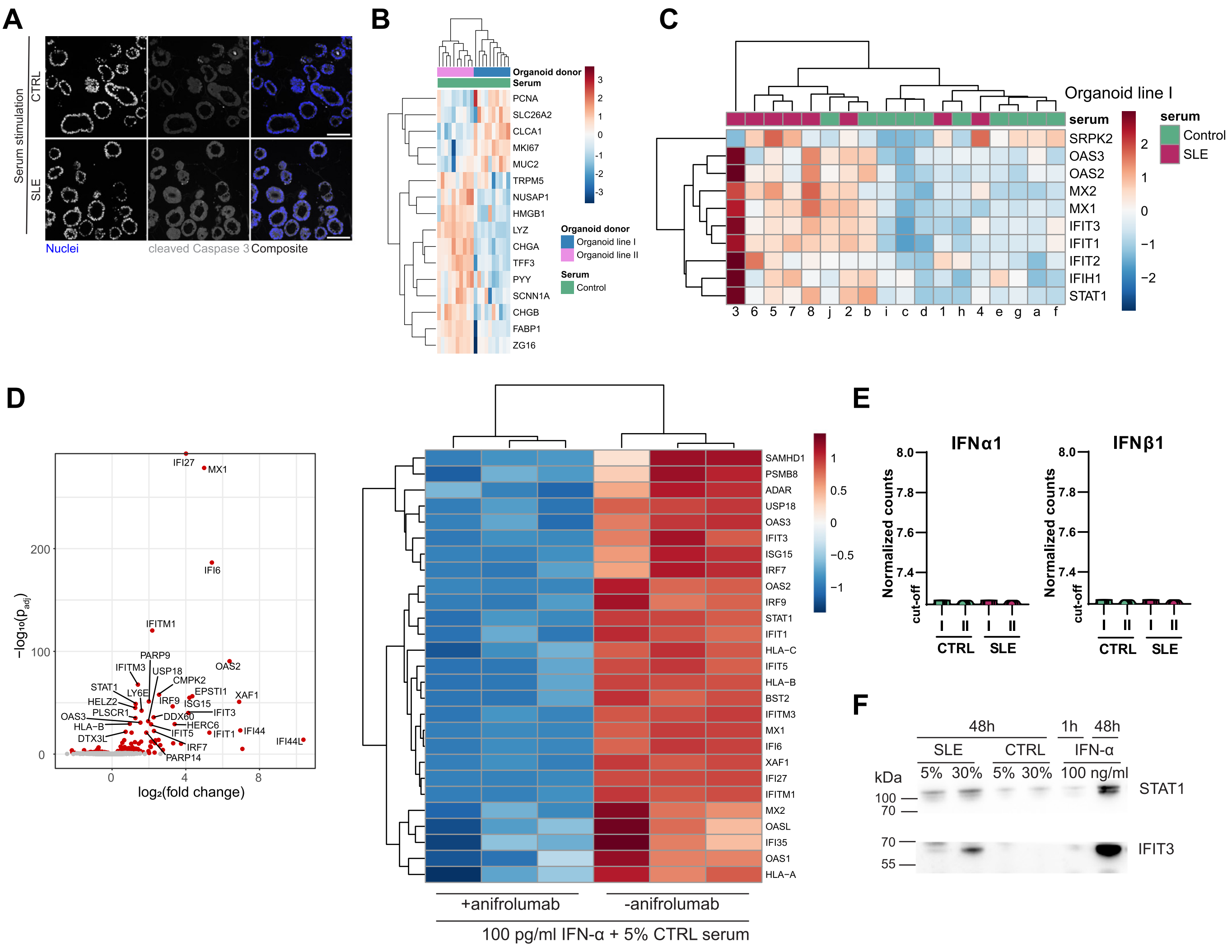

Figure EV1. Expression profile of organoids stimulated with serum or with IFN-α in combination with anifrolumab.

(**A**) Immunofluorescence of cleaved caspase 3 in either control (top) or SLE (bottom) serum-stimulated organoids. Nuclei marked by Hoechst33342 in blue and cleaved caspase 3 in gray, scale bars = 100 µm. (**B**) Heatmap with hierarchical clustering of various cell-type markers showing organoid line specific proliferation and differentiation dynamics, *n* = 10 control sera per organoid line I and II, respectively. (**C**) Heatmap with hierarchical clustering of selected IFN-inducible genes comparing SLE (*n* = 8) to control (*n* = 10) serum-stimulated organoids from organoid line I. (**D**) Volcano Plot showing DEGs (red) comparing organoids stimulated with IFN-α+control serum to additional anifrolumab treatment in organoid line II (*n* = 3 for each condition). Type I IFN-inducible genes are highlighted by the label. Corresponding heatmap with hierarchical clustering of 27 specific genes relevant in IFN-α/β signaling. (**E**) Analysis of normalized counts for IFN-α1 and IFN-α2 expression in SLE (*n* = 8) and control (*n* = 10) serum-stimulated organoids of line I and II. (**F**) Western blot analysis of STAT1 and IFIT3 expression in IFN-α, SLE and control serum-stimulated organoids after 48 h. Each sample represents two pooled wells of organoids stimulated with serum from one donor.

© The Author(s)

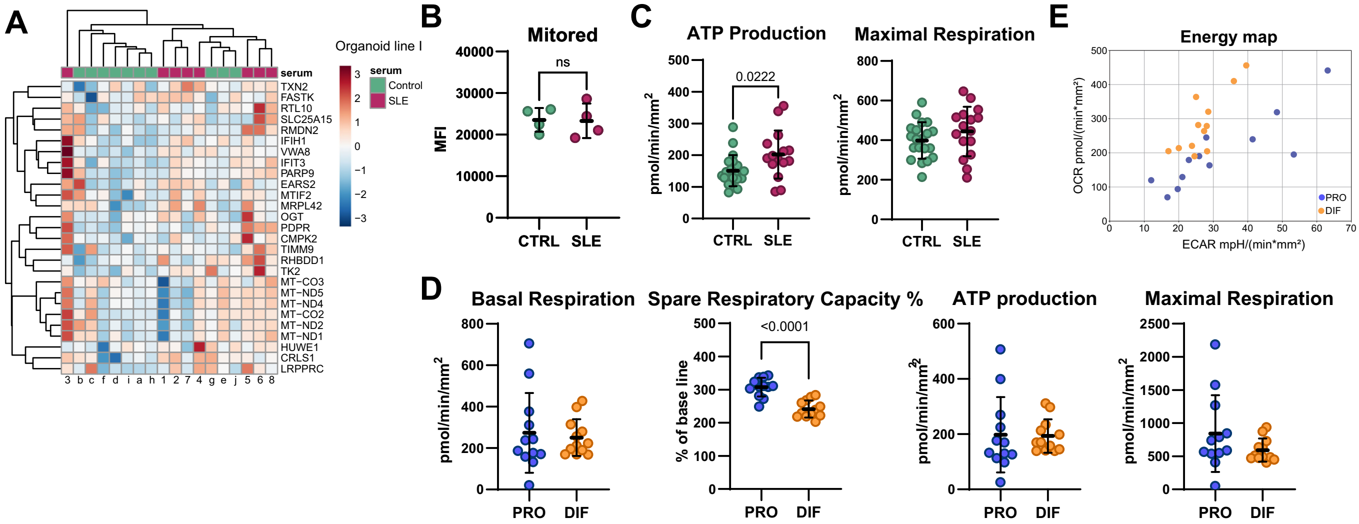

Figure EV2. Alterations of mitochondrial function are SLE serum specific.

(**A**) Heatmap with hierarchical clustering of mitochondrial genes comparing SLE to control serum-stimulated organoids from organoid line I. (**B**) Flow cytometry analysis of control and SLE serum-stimulated organoids stained with mitored. Each dot represents one well of organoids dissociated into single cells and analyzed as mean fluorescence intensity (MFI); $n = 4$ serum for each condition. Data are represented as mean ± SD, and statistical significance determined by unpaired t test. (**C**) Seahorse assay showing ATP production (left) and maximal respiration (right) comparing control (green) and SLE (pink) serum-stimulated organoids. Data are represented as mean ± SD, and statistical significance determined by unpaired t test. Each dot corresponds to one well of organoids stimulated with $n = 10$ control serum samples or $n = 8$ SLE serum samples. Each serum stimulation was analyzed with $n = 2$ technical replicates. (**D**) Seahorse assay showing basal respiration, relative spare respiratory capacity, ATP production and maximal respiration comparing organoids with a proliferative cell-type composition (PRO, blue, $n = 12$) to organoids containing additionally differentiated cells (DIF, orange, $n = 12$). Data are represented as mean ± SD, and statistical significance determined by unpaired t test. (**E**) Energy map illustrating the metabolic profile of organoids in a mainly proliferative state (blue) or additionally containing differentiated cells (orange).

© The Author(s)

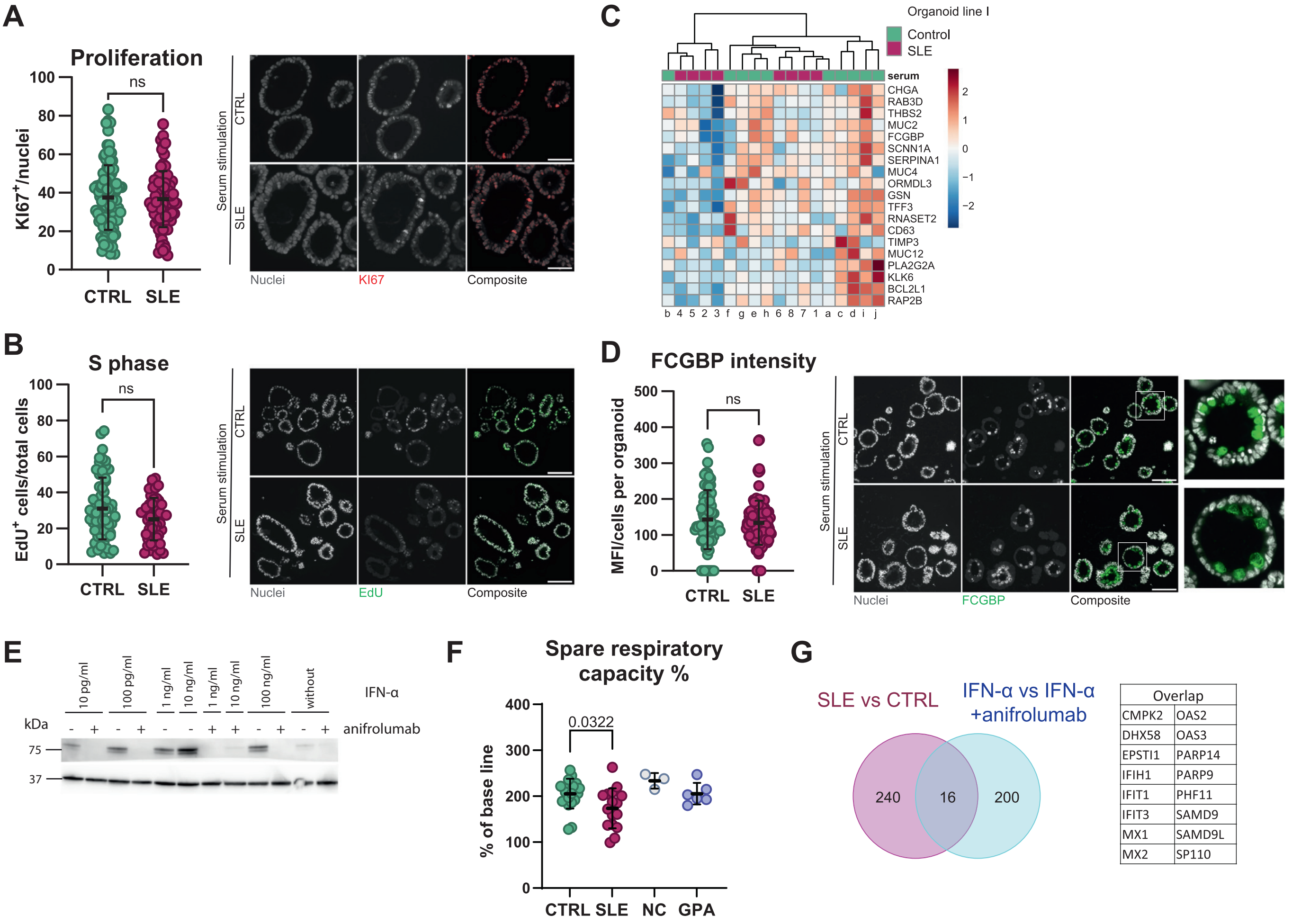

Overlap	
CMPK2	OAS2
DHX58	OAS3
EPSTI1	PARP14
IFIH1	PARP9
IFIT1	PHF11
IFIT3	SAMD9
MX1	SAMD9L
MX2	SP110

Figure EV3. Proliferation is unaltered while mitochondrial changes are distinctive to SLE serum stimulation.

(**A**) Quantification of KI67 positive cells per organoid normalized to the number of total nuclei per organoid. Each dot corresponds to a single organoid (organoid line II) stimulated with control ($n = 98$) or SLE serum ($n = 78$). Data are represented as mean ± SD, and statistical significance determined by unpaired t test. Immunofluorescence of KI67 in either control (top) or SLE (bottom) serum-stimulated organoids. Nuclei marked by Hoechst33342 in gray and KI67 in red. Scale bar = 50 µm. (**B**) Quantification of EdU (5-Ethynyl-2′-deoxyuridine) positive cells per organoid normalized to the number of nuclei per organoid. Each dot corresponds to a single organoid (organoid line II) stimulated with control ($n = 64$) or SLE serum ($n = 48$). Data are represented as mean ± SD, and statistical significance determined by Kolmogorov–Smirnov test. Immunofluorescence of EdU in either control (top) or SLE (bottom) serum-stimulated organoids. Nuclei marked by Hoechst33342 in gray and EdU in green. Scale bar = 100 µm. (**C**) Heatmap with hierarchical clustering of downregulated genes associated with the gene set "Secretory Vesicle" and "Mucus" comparing SLE to control serum-stimulated organoids from organoid line I. (**D**) Quantification of FCGBP mean intensity per organoid normalized to the number of total nuclei per organoid. Each dot corresponds to a single organoid (organoid line II) stimulated with control ($n = 79$) or SLE serum ($n = 126$). Data are represented as mean ± SD, and statistical significance determined by Kolmogorov–Smirnov test. Insets showcase enlarged areas, scale bar = 100 µm. (**E**) Western blot analysis of STAT1 phosphorylation in organoids stimulated for 48 h with increasing concentrations of IFN-α2 with or without anifrolumab treatment to validate its inhibitory effect. (**F**) Seahorse assay showing relative spare respiratory capacity comparing control (green) and SLE (pink) serum-stimulated organoids to unstimulated (NC, gray) and GPA (granulomatosis with polyangiitis) serum (violet) stimulated organoids. Data are represented as mean ± SD, and statistical significance determined by one-way ANOVA with Holm–Šidák's multiple comparisons test. Each dot corresponds to one well of organoids stimulated with control or SLE serum ($n = 10$) or GPA serum ($n = 3$). Each serum stimulation was analyzed with $n = 2$ technical replicates except NC with $n = 3$ technical replicates. (**G**) Venn diagram showing the overlap of DEGs resulting from stimulation with SLE compared to control serum and IFN-α+control serum compared to control serum. The overlapping genes are represented in the table.

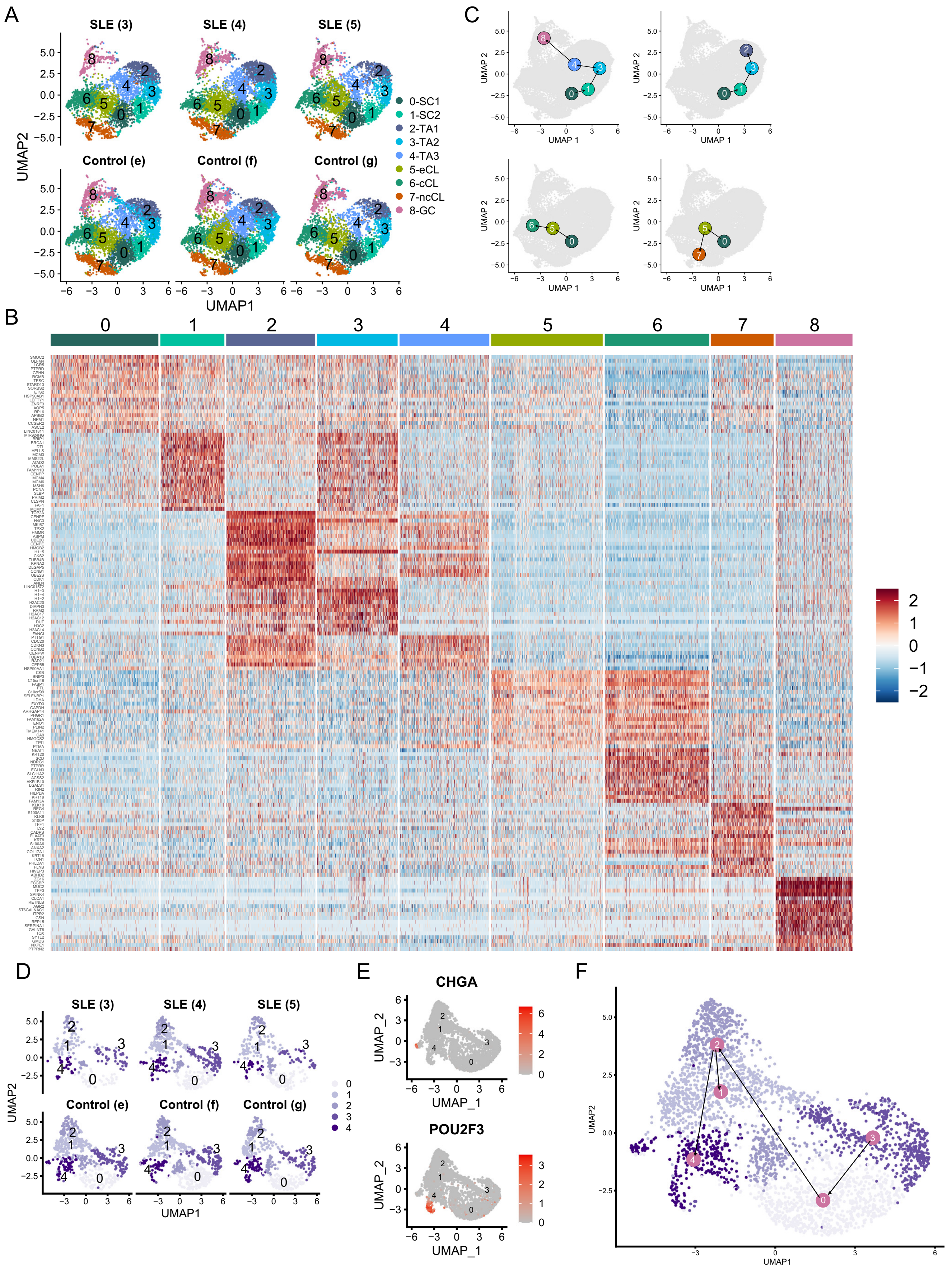
A
SLE (3)
SLE (4)
SLE (5)
Control (e)
Control (f)
Control (g)
UMAP2
UMAP1
0-SC1
1-SC2
2-TA1
3-TA2
4-TA3
5-eCL
6-cCL
7-ncCL
8-GC
C
UMAP 2
UMAP 1
B
0 1 2 3 4 5 6 7 8
2 1 0 −1 −2
D
SLE (3)
SLE (4)
SLE (5)
Control (e)
Control (f)
Control (g)
UMAP2
UMAP1
E
CHGA
POU2F3
UMAP_2
UMAP_1
F
UMAP2
UMAP1

 © The Author(s)

Figure EV4. scRNA-seq data showing sample specific clustering, heatmap and developmental trajectories.

(**A**) UMAP analysis showing the cluster distribution for each analyzed sample. (**B**) Heatmap representing the top 20 DEG per identified cluster. (**C**) UMAP plot illustrating the different predicted cellular developmental trajectory using Slingshot. (**D**) UMAP analysis showing subclustering of goblet cells for each analyzed sample. (**E**) UMAP plot illustrating the expression of CHGA and POU2F3 in goblet cell subclusters. (**F**) UMAP plot illustrating the different predicted cellular developmental trajectory within the goblet cell subcluster using Slingshot. All plots except for (**A** and **D**) show the data from all analyzed cells independent on the serum source.

© The Author(s)

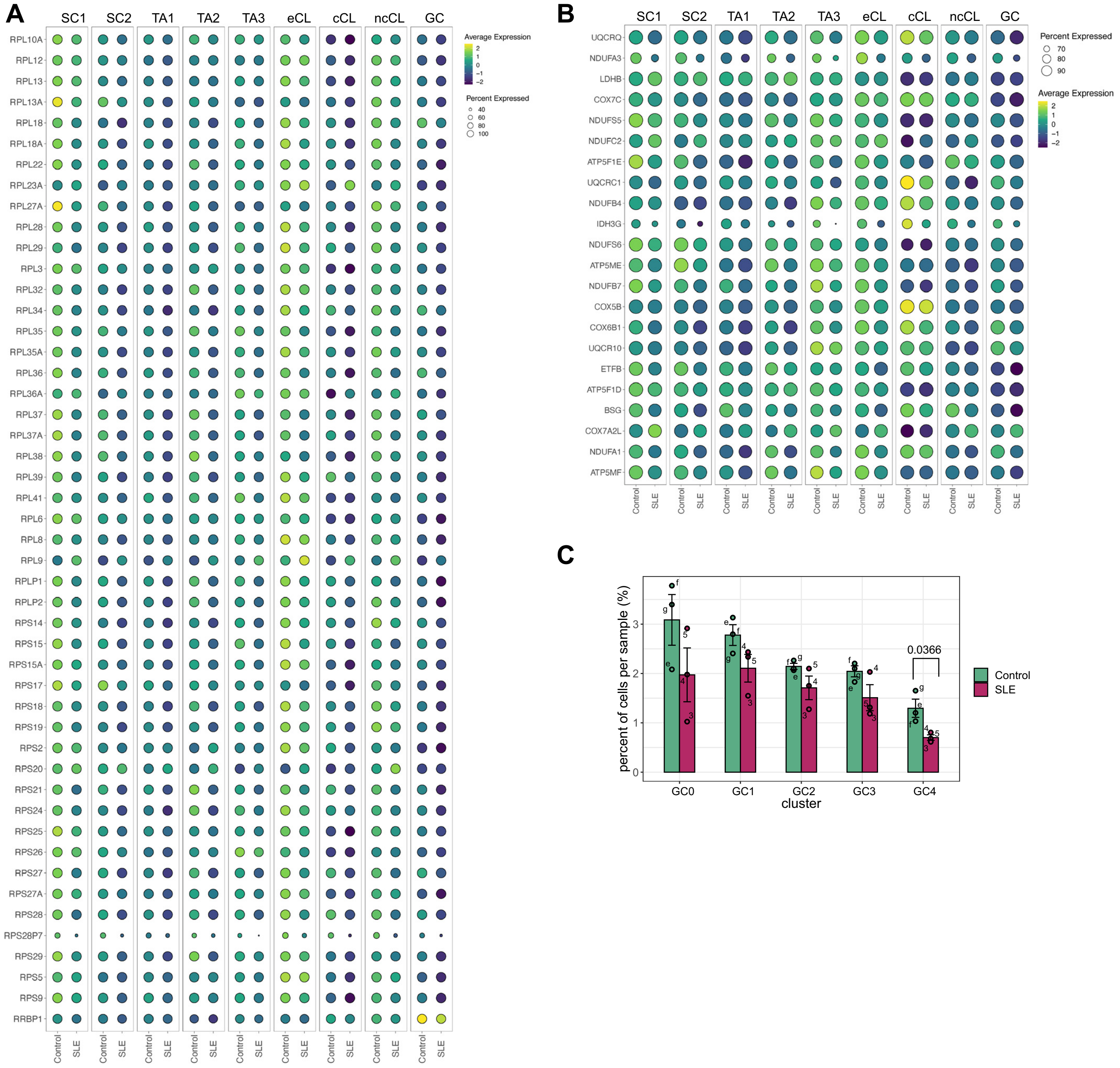

Figure EV5. Effect of SLE serum stimulation on ribosomal, mitochondrial gene expression and goblet cell subtypes.

(**A**) Dot plot representing the average expression of ribosomal genes across all identified cell types. (**B**) Dot plot representing the average expression of genes connected to oxidative phosphorylation across all identified cell types. (**C**) Analysis of the distributions of goblet cell subclusters within the total analyzed cells of SLE (pink, $n = 3$) and control (green, $n = 3$) serum-stimulated organoids. Data are presented as mean with ±SD. Unpaired *t* test was performed. (**A**, **B**) The size and color of each dot represent the cell percentage and average gene expression, respectively.

 © The Author(s)
